# Loss of tumor suppressive microRNA-31 enhances TRADD/NF-κB signaling in glioblastoma

**DOI:** 10.18632/oncotarget.4596

**Published:** 2015-06-23

**Authors:** Rajani Rajbhandari, Braden C. McFarland, Ashish Patel, Magda Gerigk, G. Kenneth Gray, Samuel C. Fehling, Markus Bredel, Nicolas F. Berbari, Hyunsoo Kim, Margaret P. Marks, Gordon P. Meares, Tanvi Sinha, Jeffrey Chuang, Etty N. Benveniste, Susan E. Nozell

**Affiliations:** ^1^ Departments of Cell, Developmental and Integrative Biology, University of Alabama at Birmingham, Birmingham, Alabama, USA; ^2^ Radiation Oncology at the University of Alabama at Birmingham, Birmingham, Alabama, USA; ^3^ Jackson Laboratory for Genomic Medicine, Farmington, Connecticut, USA

**Keywords:** NF-κB, glioblastoma, TRADD, microRNA-31

## Abstract

Glioblastomas (GBMs) are deadly tumors of the central nervous system. Most GBM exhibit homozygous deletions of the *CDKN2A* and *CDKN2B* tumor suppressors at 9p21.3, although loss of *CDKN2A/B* alone is insufficient to drive gliomagenesis. *MIR31HG*, which encodes microRNA-31 (miR-31), is a novel non-coding tumor suppressor positioned adjacent to *CDKN2A/B* at 9p21.3. We have determined that miR-31 expression is compromised in >72% of all GBM, and for patients, this predicts significantly shortened survival times independent of *CDKN2A/B* status. We show that miR-31 inhibits NF-κB signaling by targeting TRADD, its upstream activator. Moreover, upon reintroduction, miR-31 significantly reduces tumor burden and lengthens survival times in animal models. As such, our work identifies loss of miR-31 as a novel non-coding tumor-driving event in GBM.

## INTRODUCTION

Glioblastomas (GBMs) are the most common and deadly type of malignant gliomas. These tumors are highly aggressive, diffusively infiltrative, and exhibit a high degree of intra- and intertumor heterogeneity [1-4]. GBMs are characterized as Proneural (PN), Neural (N), Classical (C) or Mesenchymal (Mes) in nature, based on chromosomal number variations (CNV) [5, 6], gene expression patterns [1, 2] and abnormalities in DNA methylation [3]. However, most GBM contain more than one subtype [3], suggesting subtypes may evolve from one type into another [4, 7].

NF-κB, a family of transcription factors, mediates immune and inflammatory signaling [8]. Classical NF-κB is a cytoplasmic inactive heterodimer of p65 and p50 bound to Inhibitor of NF-κB (IκB) proteins [9]. NF-κB is activated by TNF-α and other proinflammatory cytokines. TNF-α binds to TNF Receptor 1 (TNFR1), which causes the receptor to oligomerize and recruit TNF receptor associated death domain (TRADD). TRADD recruits additional proteins that indirectly activate IκB kinase (IKK). IKK phosphorylates IκB, which promotes its degradation. Once released from IκB, NF-κB migrates to the nucleus and activates numerous target genes [9]. While NF-κB is tightly regulated, in many cancers including GBMs, NF-κB is constitutively activated, its target genes are overexpressed, and these events promote the formation and/or progression of cancer [10, 11]. As such, it is important to understand the role of NF-κB signaling in GBM.

MicroRNAs (miRs) are short, endogenous, single-stranded RNAs that inhibit gene expression [12]. MiRs are often dysregulated in cancer, which contributes to tumor development and/or progression by disrupting gene expression [13, 14]. The *MIR31HG* gene, which encodes miR-31, lies adjacent to the *CDKN2A/B* locus, the most frequently deleted loci in GBM [5, 6]. MiR-31 expression is reduced or absent in many cancers including ovarian, breast, osteosarcoma, prostate and adult T cell leukemia [15]. In GBM, the significance of miR-31 remains poorly characterized. Herein, we demonstrate *MIR31HG* is deleted in most GBM, inhibits TRADD expression, and limits NF-κB activity. Reconstitution of miR-31 inhibits the migration and proliferation of GBM cells, while loss of miR-31 significantly enhances NF-κB activity and increases tumor growth. In patients with GBM, the loss of miR-31 correlates with reduced survival times. Our data identify miR-31 as a novel non-coding tumor suppressor in GBM.

## RESULTS

### MicroRNA-31 is deleted in glioblastoma

**Figure 1:**
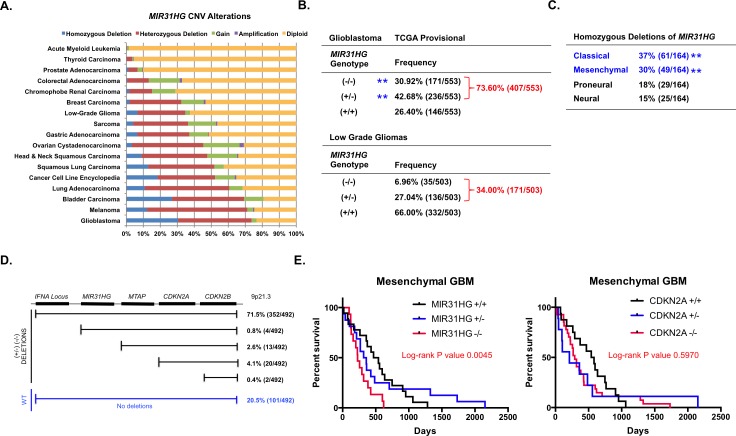
*MIR31HG* is deleted in GBM **A.** *MIR31HG* CNVs from non-embargoed cancers in the TCGA. **B.** Frequency of *MIR31HG* deletions in Glioblastoma and Low Grade Gliomas (LGG). Blue asterisks indicate that the levels of *MIR31HG* are significantly reduced compared to *MIR31HG*+/+ tumors. (**, *p* < 0.005). **C.** Homozygous *MIR31HG* deletions stratified by GBM subtype. Blue indicates significance when compared to PN and N subtypes. **D.** Diagram of chromosome 9p21.3. Indicated below are the frequencies the indicated region is deleted in GBM. **E.** Kaplan-Meier survival curve for Mes-GBM stratified by *MIR31HG* (left) or *CDKN2A* (right) genotypes.

We analyzed all cancers in the TCGA repository for status of *MIR31HG* CNVs. *MIR31HG* is deleted in numerous cancers but deletions occur most frequently in GBM, exceeding 73% of all GBMs (Figure 1A). In GBM, 30.92% were homozygous null (!−/−), 42.68% were heterozygous (+/−) and 26.40% were wildtype for miR-31 (+/+) (Figure 1B). Loss of one or both copies of *MIR31HG* significantly reduced the levels of miR-31 compared to tumors with no *MIR31HG* deletions (Figure 1B, blue asterisks; Supp. Figure 1A), indicating loss of one or both copies of *MIR31HG* is sufficient to diminish miR-31 levels in GBM. In low grade gliomas (LGG), 6.96% were *MIR31HG*^−/−^, 27.04% were *MIR31HG*^+/−^, and 66.00% were *MIR31HG*^+/+^ (Figure 1B). Chi-square analyses indicated homozygous *MIR31HG* deletions predominantly associated with Mes- and C-GBMs (Figure 1C, blue asterisks).

*MIR31HG* lies adjacent to *CDKN2B*, *CDKN2A* and *MTAP*, and the *IFNA* loci at 9p21.3 (Figure 1D). *CDKN2A* is the most frequently deleted gene in GBM [16]. We found most 9p21.3 deletions (71.5%) in GBM spanned *CDKN2B*, *CDKN2A*, *MTAP*, *MIR31HG* and the *IFNA* loci (Figure 1D). Focal deletions of 9p21.3 were present but rare (<8% of GBM). One GBM exhibited homozygous deletion of *MIR31HG* without deletions in *CDKN2A/B* (TCGA-27-1832-01) (Supp. Figures 1B, 1C). Nearly 21% of all GBM harbor no deletions at 9p21.3. We also found miR-31 expression was absent in established human GBM cell lines (Supp. Figure 1D, indicated by “-“) and human GBM xenografts (Supp. Figure 1E, indicated by “-“).

### *MIR31HG* deletions predict poor prognosis in human GBM

We used the Mantel-Cox test to assess the significance of homozygous deletions of *CDKN2A* or *MIR31HG* on overall patient survival (Median Months Survival (MMS), left 2 columns) and Disease Free Survival (DFS) (median months disease free, right two columns) in GBM (). *CDKN2A* deletions were associated with diminished DFS times in all GBM, and patients with N-GBM. *MIR31HG* deletions predicted shorter MMS in patients with primary GBM (Supp. Figure, Table I) and patients with Mes-GBM (Figure 1E, left). Importantly, deletions in *CDKN2A*, which lies adjacent to MIR31HG, did not predict shorter MMS in patients with Mes-GBM (Figure 1E, right) or primary GBM (Supp. Figure, Table I). *MIR31HG* deletions were not correlated with diminished DFS (Supp. Figure, Table I).

**Table 1:** Patient data from GBMs stratified according to *CDKN2A* or *MIR31HG* Status

	(Log-rank)			(Log-rank)			(Log-rank)			(Log-rank)		
GROUP	P-VALUE	MEDIAN MONTHS SURVIVAL		P-VALUE	MEDIAN MONTHS SURVIVAL		P-VALUE	MEDIAN MONTHS DISEASE FREE		P-VALUE	MEDIAN MONTHS DISEASE FREE	
		*CDKN2A* WT	*CDKN2A* Null		*MIR31HG* WT	*MIR31HG* Null		*CDKN2A* WT	*CDKN2A* Null		*MIR31HG* WT	*MIR31HG* Null
All GBM (n=598)	0.055366	14.93	13.91	0.217208	14.20	12.92	0.029511	8.02	6.61	0.344599	7.33	6.18
Primary GBM (n=515)	0.056588	13.87	12.92	0.049266	18.87	12.16	0.17803	7.00	6.18	0.095283	6.97	6.02
Recurrent GBM (n=27)	0.440135	26.99	19.86	0.795598	22.72	48.95	0.884115	14.79	11.77	0.442442	14.79	14.04
Classical (n=146)	0.666358	15.95	14.50	0.269566	15.35	14.47	0.109498	9.63	7.33	0.193350	7.82	7.82
Mesenchymal (n=157)	0.178072	12.95	11.67	0.043361	12.95	10.45	0.791568	6.67	5.65	0.233200	6.67	5.65
Neural (n=83)	0.457480	14.93	14.53	0.763517	13.97	15.65	0.043853	14.04	8.52	0.612740	8.52	10.19
Pro-Neural (n=99)	0.500123	10.59	9.53	0.473928	10.59	7.82	0.426399	5.39	4.31	0.783452	5.39	2.99
G-CIMP (n=42)	0.861556	33.67	47.11	0.751448	33.67	82.55	0.717234	15.19	16.54	0.530118	16.54	34.62
Non-G-CIMP (n=490)	0.177029	12.95	12.92	0.187257	13.28	12.16	0.062134	6.84	6.18	0.327952	6.84	6.12
MGMT Methylated (n=170)	0.132838	17.75	16.50	0.240705	17.75	16.50	0.132964	10.09	8.45	0.935303	9.01	8.68
MGMT Unmethylated (n=181)	0.532280	14.73	12.23	0.262724	12.95	14.53	0.607534	6.02	7.33	0.153410	6.71	6.67
IDH WT (n=392)	0.189003	14.70	14.14	0.470236	14.14	13.91	0.056167	8.35	6.67	0.639004	6.71	7.17
IDH1 MT (n=31)	0.813419	33.67	82.55	0.769843	33.67	82.55	0.391472	11.54	8.55	0.391472	11.54	8.55

### MiR-31 expression inversely correlates with TRADD expression

**Figure 2:**
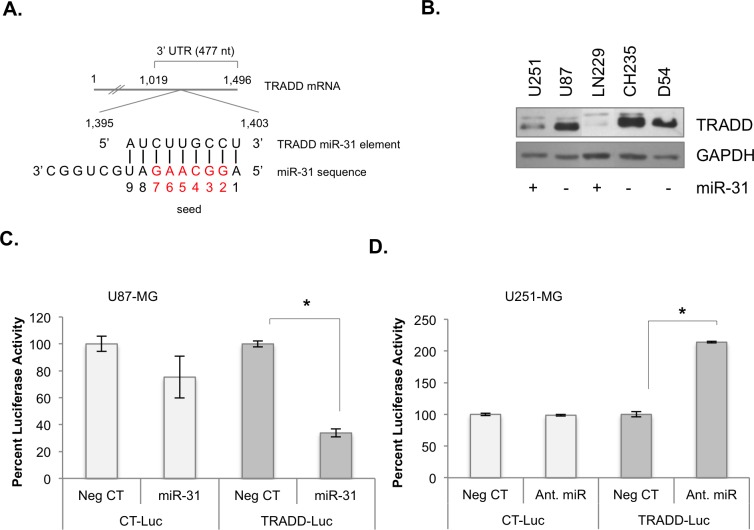
*TRADD* is a target of miR-31 **A.** Diagram depicting *TRADD* mRNA, and its putative miR-31 seed sequence (red). **B.** The levels of miR-31 and TRADD protein in human glioma cell lines of known miR-31 status. **C.** U87-MG cells were transfected with luciferase expression plasmid containing a control 3′ UTR (CT-Luc) or the 3′ UTR of *TRADD* (TRADD-Luc), and either control miR (Neg CT) or miR-31, and luciferase activity was analyzed. (*, *p* < 0.05). **D.** U251-MG cells were transfected with either CT-Luc or TRADD-Luc, and then with either Neg CT or antagomiR-31. Luciferase activity was analyzed. (*, *p* < 0.05).

In GBM, TRADD is overexpressed (Supp. Figure 2) and NF-κB is constitutively activated [10, 11, 17-19], particularly in Mes-GBM [3]. We identified a consensus miR-31 binding element within the *TRADD* 3′ UTR (Figure 2A). In established malignant glioma cells, TRADD levels were inversely correlated with miR-31 status (Figure 2B). We used the luciferase reporter assay to assess the validity of the *TRADD* miR-31 element. U87 miR-31 (™) cells were transfected with negative control miRNA (Neg CT) or miR-31 for 48 h and then transfected with TRADD-Luc, which contains the TRADD 3′ UTR, or a control plasmid (CT-Luc) for 24 h. MiR-31 significantly inhibited luciferase activity in cells transfected with TRADD-Luc, but not in cells transfected with CT-Luc (Figure 2C). AntagomiRs are chemically modified oligonucleotides that bind to and inhibit the functions of miRs [20]. U251 miR-31 (+) cells were transfected with Neg CT or antagomiR-31 for 48 h and the plasmids described above. AntagomiR-31 significantly increased luciferase activity in cells containing TRADD-Luc, but not in cells containing CT-Luc (Figure 2D). These data indicate that the *TRADD* miR-31 element is sensitive to miR-31.

### MiR-31 regulated TRADD inhibits NF-κB signaling

Next, we assessed the impact of miR-31 on TRADD expression and NF-κB activation. We found U87-MG miR-31 (™) cells expressed significantly more TRADD RNA and protein than U251-MG miR-31 (+) cells, and this was not altered by TNF-α stimulation (Supp. Figures 3A-3C). Additionally, the levels of activated NF-κB (Supp. Figure 3D), and its target genes *I*κ*B*α (Supp. Figure 3E), *IL-6*, *IL-8*, *A20*, *SOCS3* and *c-Myc* (Supp. Figure 3F) were significantly greater in U87 cells compared to U251 cells. Herein, we chose to use the *IL-6* gene as a reporter of miR-31 mediated effects on TRADD/NF-κB signaling. As shown in Supp. Figures. 3G and 3H, U87-MG cells expressed higher levels of IL-6 mRNA and protein in response to TNF-α stimulation compared to U251-MG cells.

**Figure 3:**
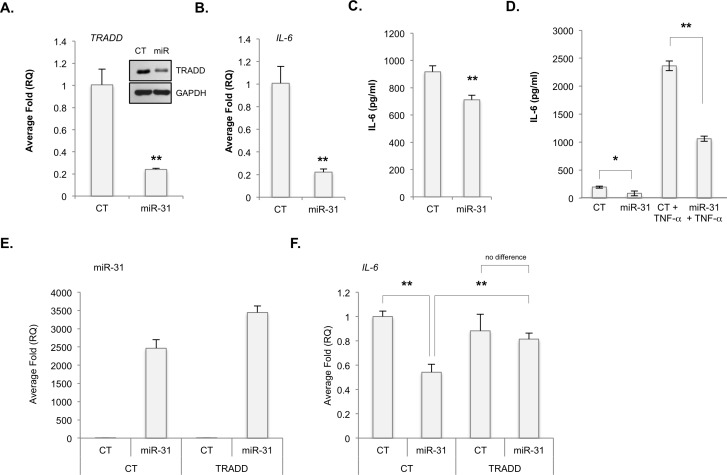
MiR-31 inhibits TRADD expression and NF-κB-induced gene expression **A.**-**D.** U87-MG cells were transfected with control (CT) miR or miR-31. A. TRADD mRNA and protein levels were evaluated by qRT-PCR and immunoblot analyses, respectively. (**, *p* < 0.005). **B.**, **C.** The levels of IL-6 mRNA **B.** and protein **C.** in U87-MG cells as described in **A.**, were evaluated by qRT-PCR and ELISA, respectively. (**, *p* < 0.005). **D.** U87-MG cells were transfected with CT or miR-31, and grown in the absence or presence of TNF-α (10 ng/ml). IL-6 protein levels were evaluated by ELISA. (*, *p* < 0.05; **, *p* < 0.005). **E.**, **F.** U87-MG cells were transfected with CT or miR-31, and either a control plasmid (CT) or a plasmid expressing TRADD, but lacking the 3′ UTR. MiR-31 **E.** and IL-6 **F.** RNA levels were measured by qRT-PCR. (**, *p* < 0.005).

**Figure 4:**
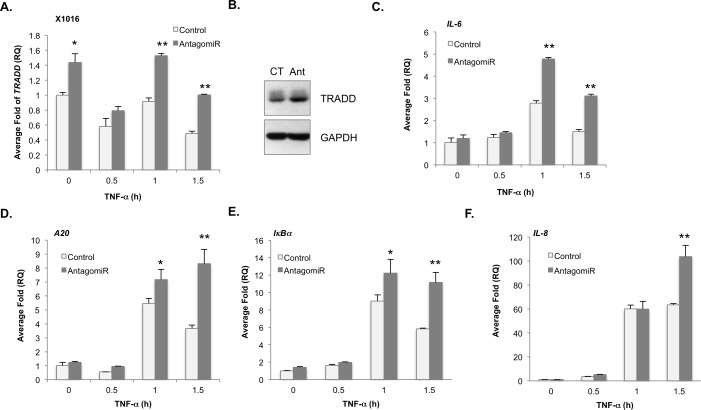
Loss of miR-31 enhances *TRADD* and NF-κB target gene expression in human GBM xenografts **A.**, **B.** Human GBM xenograft X1016 was transfected with control or AntagomiR-31 (AntagomiR) and the levels of *TRADD* mRNA **A.** and protein **B.** evaluated by qRT-PCR or immunoblotting. (*, *p* < 0.05; **, *p* < 0.005). **C.**- **F.** The levels of *IL-6* mRNA **C.**, *A20* mRNA **D.**, *I*κ*B*α mRNA **E.** and *IL-8* mRNA **F.** in X1016 cells transfected as described in (A) were evaluated by qRT-PCR. (*, *p* < 0.05; **, *p* < 0.005).

Next, U87-MG cells were transfected with either a control miRNA (CT) or a miR-31 mimic (miR-31). MiR-31 reduced the levels of TRADD mRNA and protein (Figure 3A), and IL-6 mRNA and protein (Figure 3B, 3C, 3D). We confirmed miR-31 inhibited E2F2 (Supp. Figure 4), a validated target of miR-31 [21, 22]. Above, our data suggest that miR-31 inhibits TRADD and consequently NF-κB signaling. To test this, we assessed whether TRADD overexpression would rescue NF-κB signaling. U87 cells were first transfected with CT or miR-31 for 48 h. Next, cells were transfected with either a control plasmid (CT) or a plasmid encoding TRADD. As the TRADD plasmid lacks the 3′ UTR, it restores TRADD expression but is insensitive to miR-31. We found miR-31 reduced the levels of IL-6, but not if TRADD were co-expressed (Figure 3F). These data were also confirmed in U251-MG cells (Supp. Figure 5) and in patient-derived GBM xenograft cells (PDGXs). X1016 miR-31 (+) cells transfected with miR-31 antagomiR displayed higher levels of *TRADD* mRNA (Figure 4A) and protein (Figure 4B) than did X1016 cells transfected with CT miRNA. Finally, the levels of the NF-κB regulated genes *IL-6* (Figure 4C), *A20* (Figure 4D), *I*κ*B*α (Figure 4E) and *IL-8* (Figure 4F) were higher in cells with miR-31 antagomiR compared to cells transfected with CT miRNA. These data confirm that miR-31 limits NF-κB signaling by regulating TRADD expression.

### *MIR31HG* and NFKBIA deletions are mutually exclusive in GBM

NF-κB is constitutively activated in GBM [10, 11, 17, 18]. Mono-allelic deletions in *NFKBIA*, which encodes an inhibitor of NF-κB, are predominantly found in Non-C-GBM[11]. We found *MIR31HG* deletions also enhanced NF-κB activation (Figures 3, 4), but this event predominated in C- and Mes-GBM (Figure 1C). Therefore we reasoned that *NFKBIA* and *MIR31HG* deletions might stratify to distinct GBM subtypes. We found *NFKBIA* deletions were mutually exclusive with *MIR31HG* deletions (*p* = 0.0029) (Supp. Figure 6A), but not with deletions of a gene adjacent to *MIR31HG* (*CDKN2A*) (Supp. Figure 6B). These data suggest that there may be selective pressure to delete *MIR31HG* in GBMs that do not harbor mono-allelic *NFKBIA* deletions as these CNVs may be functionally redundant with respect to NF-κB activation.

### NF-κB induces miR-31

**Figure 5:**
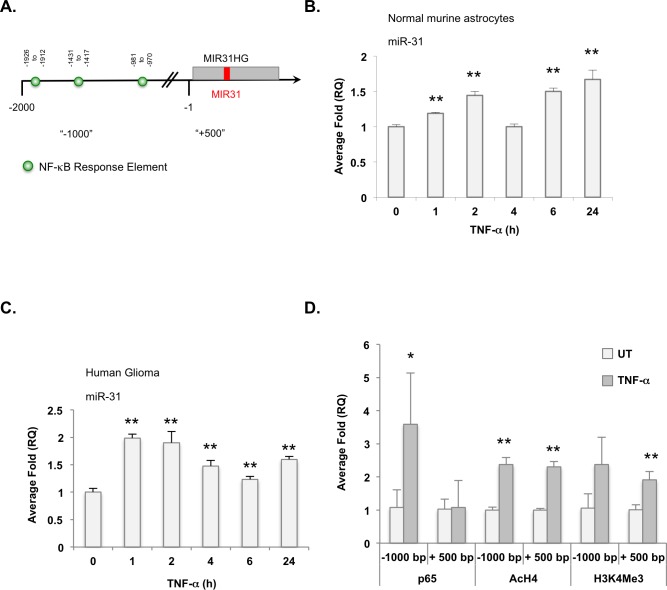
TNF-α activated NF-κB induces miR-31 expression **A.** Diagram of the *MIR31HG* promoter and putative NF-κB response elements. Positions marked are relative to transcriptional start site. **B.**, **C.** Normal murine astrocytes **B.** or U251-MG cells **C.** were stimulated with TNF-α (10 ng/ml) for the times indicated and miR-31 levels evaluated by qRT-PCR. (**, *p* < 0.005). **D.** U251-MG cells were left untreated (UT) or stimulated with TNF-α (10 ng/ml) for 4 h, and ChIP analyses were performed using the antibodies specified. Primers targeting regions upstream (“-1000”) and downstream of the TSS (“+500”) were used. (*, *p* < 0.05; **, *p* < 0.005).

Because NF-κB regulates IκBα, its own inhibitor, we hypothesized that NF-κB also regulates miR-31 expression. Indeed, the *MIR31HG* promoter [21, 23] contains three putative NF-κB binding sites (Figure 5A). To assess whether NF-κB induces miR-31, normal murine astrocytes were grown in the absence or presence of TNF-α for various times and miR-31 levels were measured. In response to TNF-α stimulation, miR-31 expression levels were elevated and displayed an oscillation pattern consistent with NF-κB activation patterns[24, 25] (Figure 5B). These data were confirmed in human glioma cells where miR-31 levels were elevated in response to TNF-α stimulation (Figure 5C). We used chromatin immunoprecipitation (ChIP) assays to assess whether TNF-α induced miR-31 expression required NF-κB. In the absence of TNF-α, minimal NF-κB p65 binding was detected at the *MIR31HG* promoter (Figure 5D). After TNF-α stimulation, the levels of NF-κB p65 were significantly elevated, but only at the distal −1000 bp site, which contains an NF-κB response element. We also evaluated acetylated histone 4 (AcH4) and tri-methylated histone 3 at lysine 4 (H3K4Me3) levels as mark areas of active transcription [26]. In resting cells, the *MIR31HG* promoter exhibits low levels of AcH4 and H3K4Me3, but these levels were significantly increased upon TNF-α stimulation (Figure 5D). These data indicate that NF-κB induces the expression of miR-31, likely to limit its own activities.

### MiR-31 inhibits the migration and growth of GBM cells *in vitro*

Next, we assessed the functional effects of miR-31 in glioma cells. We analyzed cell migration using U251-MG cells transfected with either CT or AntagomiR. Cells were grown to 100% confluence and then wounded (initial percent open area = ~30%) (Supp. Figure 7A, Initial). After 8 h, ~16% of the wound remained present in cells transfected with CT, while cells with reduced miR-31 expression displayed only ~4% of the wound at 8 h (Supp. Figure 7A, Final), indicating miR-31 inhibits cell migration. To evaluate the impact of miR-31 on GBM cell growth, we used the WST-1 assay on U87-MG cells transfected with CT or miR-31. Glioma cells expressing miR-31 displayed reduced cell growth compared to cells transfected with CT (Supp. Figure 7B).

### MiR-31 inhibits GBM growth *in vivo*

**Figure 6:**
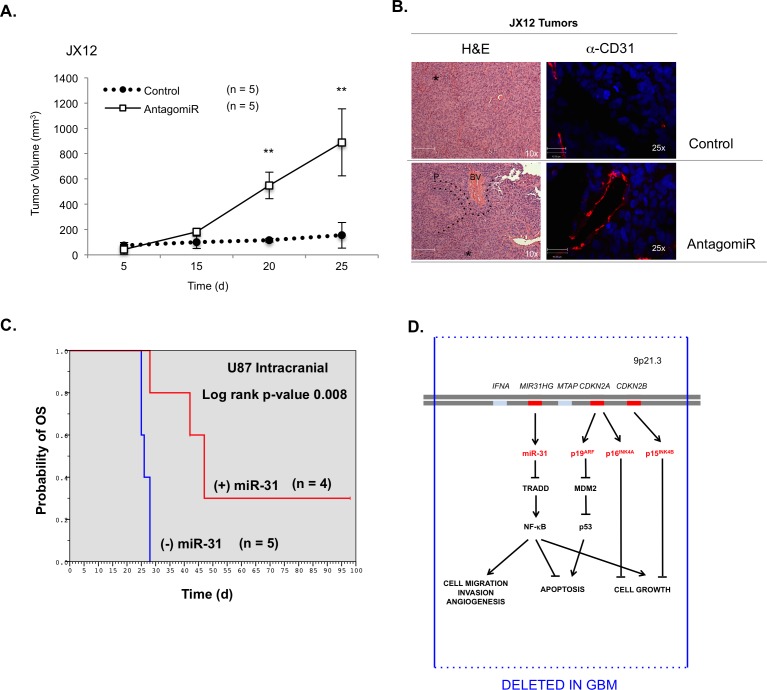
The levels of miR-31 inversely correlate with tumor growth *in vivo* **A.** Human GBM xenograft JX12 was transfected with control or AntagomiR-31 (AntagomiR) and injected into the flanks of nude mice (*n* = 5) and volume measured. (**, *p* < 0.005). **B.** Mouse bearing JX12 miR-31 positive tumor (green circle) and JX12 miR-31 reduced tumor (red circle). Tumors resections were analyzed with H&E staining (x, y-scale of 42 μm). Blood vessel formation indicated with BV, and areas of necrosis are indicated with an (*). Blood vessels were stained using α-CD31 antibodies (x, y-scale of 42 μm). **C.** Stable U87-MG cells that express CT miR (™) or miR-31 (+) were injected intracranially, and survival times recorded as probability of overall survival (OS). **D.** Chr 9p21.3 is deleted in most GBM. This locus harbors three tumor suppressor genes (red lines), which encode four tumor suppressors (red type) in GBM: miR-31, p16^INK4A^, p19^ARF^ and p15^INK4B^. Loss of these genes increases cell migration, invasion, angiogenesis and cell growth, and apoptosis is inhibited.

To assess the impact of miR-31 on tumor growth, we used JX12 PDGXs, which are characterized as Classical GBM and are miR-31 (+). These cells were transfected with a control miRNA (CT) or antagomiR-31 *ex vivo* for 48 h, and then injected s.c. and tumor growth was monitored. JX12 cells with reduced miR-31 expression grew faster and larger than JX12 tumors expressing miR-31 (Figure 6A & 6B). Tumors with reduced miR-31 levels (AntagomiR) appeared heterogeneous, showed blood vessel (BV) formation (Figure 6B, 6H & 6E, α-CD31), and expressed higher levels of TRADD and activated NF-κB (P-p65) (Supp. Figure 8A). Additionally, tumors with antagomiR-31 showed elevated levels of genes associated with NF-κB activation (IL-6), angiogenesis (VEGF), proliferation (Cyclin D1) and Mesenchymal GBMs (TAZ, YKL40), and reduced levels of E-cadherin, which correlates with epithelial to mesenchymal transition (EMT) (Supp. Figure 8B). These data were confirmed using an intracranial GBM model, wherein the loss of miR-31 in Classical GBM correlated with a switch to a more Mesenchymal GBM profile (Supp. Figures 8C).

We repeated these experiments using stable U87 cells that express CT miR (-miR-31) or miR-31 (+miR-31) or U251 cells that express CT (+ miR-31) or miR-31 sponge, which inhibits miR-31 function (-miR-31). At the end of these experiments, cells with no or reduced miR-31 formed more tumors (3/5 in U87, 4/5 in U251) than cells with miR-31 (0/5 in U87, 2/5 in U251) (Supp. Figure 9A). Unfortunately, tumors formed at different rates, making parallel measurements difficult. However, at euthanasia, tumors with no or reduced miR-31 tended to be larger than tumors expressing miR-31 (Supp. Figure 9B). H&E analyses indicated that U251 cells with reduced miR-31 activity had increased cellularity and showed BV formation (Supp. Figure 9C, α-CD31).

### MiR-31 prolongs survival *in vivo* in an intracranial GBM model

Finally, we evaluated the role of miR-31 on intracranial GBM growth in mice using stable U87-MG cells expressing CT (- miR-31) or miR-31 (+ miR-31). Within 30 days, all mice with (™) miR-31 cells were moribund and had to be euthanized (Figure 6C). However, mice receiving (+) miR-31 cells survived significantly longer (*p* = 0.008) (Figure 6C). These data indicate that restoring miR-31 expression inhibits GBM growth and prolongs survival *in vivo*. Upon resection, we assessed miR-31 levels (Supp. Figure 10A), and confirmed that tumors with miR-31 cells displayed lower levels of TRADD and activated P-p65 compared to tumors lacking miR-31 (Supp. Figure 10B). We also confirmed that miR-31 inhibited E2F2 (Supp. Figure 10C), which regulates cell growth and is inhibited by miR-31 [21, 22].

## DISCUSSION

Herein we characterize miR-31 as a novel tumor suppressor encoded by the *MIR31HG* gene. *MIR31HG* is dysregulated in many cancers but *MIR31HG* deletions are most profound in GBM (>73% all GBM). *MIR31HG* is situated at 9p21.3, adjacent to *CDKN2A*, and statistically, *MIR31HG* deletions coincide with *CDKN2A* deletions (*p* < 0.005). Most studies have focused on the consequences of *CDKN2B* and *CDKN2A* deletions, and rarely acknowledge the impact of other deletions at 9p21.3. However, we find nearly all deletions at 9p21.3 impact *CDKN2B*, *CDKN2A*, and *MIR31HG*. Using GBM cells and PDGXs, we find that altering the levels of endogenous miR-31 alone is sufficient to significantly alter GBM behavior *in vitro* and *in vivo*. Finally, we show that loss of *MIR31HG* predicts poor prognosis in patients with primary GBM and/or Mes-GBM. Based on our findings, we propose *MIR31HG* is a novel non-coding tumor suppressor gene in GBM (Figure 6D).

We believe miR-31 suppresses tumor formation, in part, by targeting TRADD to reduce NF-κB activation. Our studies focus on NF-κB for several reasons. NF-κB is a critical mediator of inflammation, which has been linked to all phases of tumor development. We and others show that NF-κB is constitutively activated in GBM and correlated with poor patient prognosis [10, 17, 27]. Additionally, NF-κB induces the expression of proteins (IL-6, PDGFB, EGFR, HGF), which activate pathways (STAT3, PDGFR, EGFR, MET) and processes (angiogenesis, migration, invasion) critical for GBM maintenance [28]. Finally, as Mes-GBM are characterized by elevated levels of TRADD and NF-κB activity [1, 2, 7, 29], the loss of miR-31 may explain, in part, the consequences of activated NF-κB in this GBM subtype. For example, NF-κB induces IL-6 expression to ensure the activation of STAT3 [30], a master regulator of Mesenchymal GBMs [31, 32]. However, we acknowledge that additional, previously identified targets of miR-31 (E2F2 and radixin) as well as the loss of additional genes at 9p21.3 also impact GBM growth [33-35].

In this study, we demonstrate that NF-κB induces miR-31 expression and that in turn, miR-31 inhibits the levels of TRADD, an upstream activator of NF-κB. Therefore, we propose that the degree of NF-κB activation is regulated, in part, by the relationship between TRADD/NF-κB and miR-31. Unfortunately in GBM, NF-κB is constitutively activated, perhaps as a consequence of loss of proper regulation. Indeed, *MIR31HG* and/or *NFKBIA* are frequently deleted, while ING4, which inhibits NF-κB, is mutated or absent [10, 11, 17], and Pin1, an enhancer of NF-κB activity, is often overexpressed [19]. Consequently, GBMs utilize numerous mechanisms to ensure constitutive NF-κB activation.

MicroRNA-mediated therapy to inhibit NF-κB in GBM is enticing for several reasons. In particular, miRs readily cross cell membranes [36] and the blood-brain barrier[37]. Moreover, miRs are not immunogenic, and because they lack poly-A tail, these molecules are fairly stable both *in vitro* and *in vivo* [38, 39], although they can be further modified to prolong their biological half-life [40]. Presently, numerous mechanisms to restore tumor suppressive miR expression are being considered, including cationic lipid complexes, magnetic lipid complexes and viral hosts [36, 41]. Therefore, we are hopeful that this study provides enticing data to support the use of miRs, specifically miR-31, for the treatment of GBM and other cancers.

## MATERIALS AND METHODS

### Malignant glioma cell lines and human GBM xenografts

Established human glioma cell lines were obtained from ATCC and maintained as previously described [10]. Patient derived GBM xenografts (PDGXs) were maintained as previously described [30]. The UAB Brain Tumor Animal Model Core Facility performed short tandem repeat DNA profiling on all human glioma cell lines and xenografts.

### *In silico* data

Data was obtained from The Cancer Genome Atlas (TCGA) pilot project established by the NCI and NHGRI. Information about TCGA and the investigators and institutions who constitute the TCGA research network can be found at http://cancergenome.nih.gov/. Analyses were performed using cbioportal, Oncomine and microrna.org, and all references therein [42, 43]. The TCGA Provisional data set was analyzed using “miR-31/31” “MIR31HG” or “MIR31” as a search parameter.

### Reagents

TNF-α was from R & D Systems. Secondary antibodies and enhanced chemiluminescence reagents were from Amersham. Anti-TRADD and anti-p65 antibodies were from Santa Cruz Biotechnology. Anti-phosphorylated p65 (p-p65) and anti-AcH3 antibodies were from Cell Signaling, and anti-GAPDH and anti-H3K4Me3 antibodies were from Abcam. Real time qRT primers and Taqman qRT-PCR reagents were from Applied Biosystems. Sybr green qRT-PCR reagents were from Qiagen. MicroRNA-31 mimic, miR-31 antagomiR and negative control (−CT) miR were purchased from Ambion/Life Technologies. MicroRNAs were transfected using Lipofectamine RNAiMax Transfection reagent (Life Technologies).

### Scratch wound assay

Scratch wound assays were performed as previously described [19] and quantitated using Tscratch software [44].

### *In vivo* tumor models, tumor fixation, processing and immunofluorescence

Tumor injections were performed as previously described [30, 45]. In s.c. flank experiments, JX12, U87-MG or U251-MG cells (5 × 10^5^) were injected into the flanks of athymic nude mice. Tumors were measured on the indicated days using digital calipers and tumor volume was calculated using the following equation: v = (0.5 × longest diameter × shortest diameter^2^). In intracranial GBM experiments, U87 cells (5 × 10^5^) or JX12 cells (3 × 10^5^) in 5 μl of methylcellulose were injected 2 mm anterior and 1 mm lateral to bregma at a depth of 2 mm over 2 minutes as previously described [30, 45]. Mice were monitored for survival. After euthanasia, tumors were excised and snap frozen for RNA, miRNA, protein and/or IHC analyses.

### Statistical analysis

Student *t* tests, Mann-Whitney rank sum tests, Mantel-Cox tests, and LogRank tests for Kaplan-Meier survival curves were used as previously described [11, 46]. *p* < 0.05 was considered statistically significant.

### Ethics statement

*In vivo* experiments in female athymic nude mice were performed with the approval of UAB IACUC.

## SUPPLEEMENTARY MATERIAL FIGURES


