# Supplementary material for: Loss of tumor suppressive microRNA-31 enhances TRADD/NF-κB signaling in glioblastoma
Source: Oncotarget. 2015 Jun 23;6(19):17805–16. doi: 10.18632/oncotarget.4596 (PMC4627347; doi:10.18632/oncotarget.4596)
Supplement: Supplementary file 1 [file oncotarget-06-17805-s001.pdf]

Loss of tumor suppressive microRNA-31 enhances TRADD/NF-κB signaling in glioblastoma

Supplementary Material

A. B.

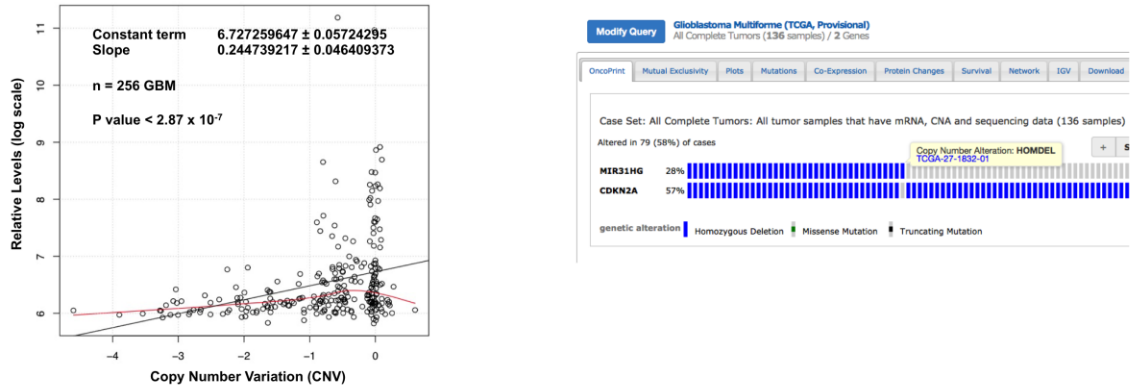

C.

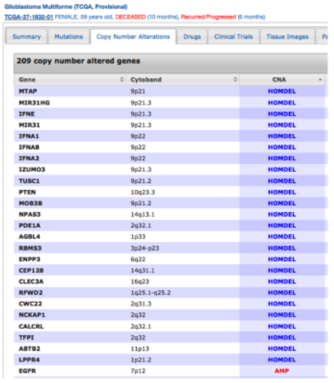

D.

Est. Cell Line	*miR-31*	*MIR31HG*	*CDKN2A*
U87-MG	-	-/-	-
CH235-MG	-	n/a	n/a
D54-MG	-	n/a	n/a
U118-MG	-	-/-	-
LN319-MG	+	n/a	+
U251-MG	+	+/?	-
LN229-MG	+	+/?	-
U373-MG	+	+/?	-

n/a : data not available

E.

Xenograft	*miR-31*	*CDKN2A*
X1046	-	-
JX14	-	-
JX22	-	-
JX12	+	-
JX39	+	-
X1016	+	-
X1066	+	+

Supp. Fig. 1. *MIR31HG* is Deleted or Reduced in Human GBM. A. Linear regression assessing the relationship between gene dosage and expression for miR-31 in 256 GBM of the TCGA set, using expression as the outcome and gene dosage as the predictor. Scatterplots and LOWESS smooths were used to confirm the suitability of linear regression analysis, and statistical relationship was assessed according to the P value for the estimated slope of the regression line. **B, C.** GBM Sample TCGA-27-1832-01 exhibits homozygous deletions in *MIR31HG* but not *CDKN2A*. **C.** Complete deletion report for TCGA-27-1832-01. **D, E.** Levels of miR-31 transcript were assessed and samples scored as (-) or (+) when compared to control.

Genomic status of *MIR31HG* gene was assessed using the Cancer Cell Line Encyclopedia. -/-, homozygous *MIR31HG* deletion, +/?, cells harbor at least one *MIR31HG* allele. The genomic status of *MIR31HG* in human GBM xenografts was not evaluated. n/a denotes data not available.

TRADD Expression in TCGA Brain

Brain Glioblastoma vs. Normal

**Over-expression Gene Rank:**	2055 (in top 17%)	**P-value:**	2.14E-6
**Reporter:**	205641_s_at	**t-Test:**	7.217
		**Fold Change:**	1.269

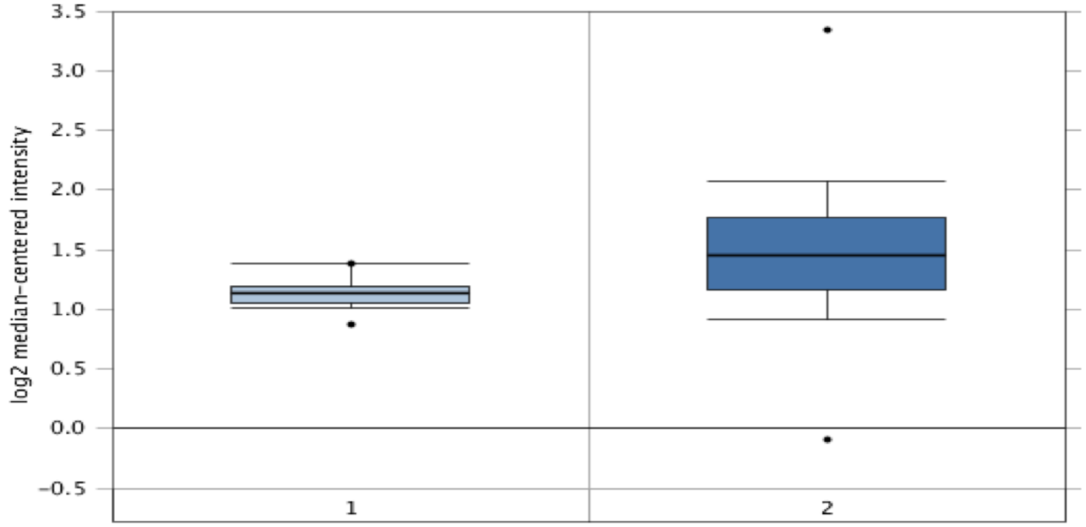

Legend

1. Brain (10)
2. **Glioblastoma (542)**

Supp. Fig. 2. ***TRADD*** **mRNA is Significantly Elevated in GBM.** *TRADD* mRNA levels were assessed using Oncomine.

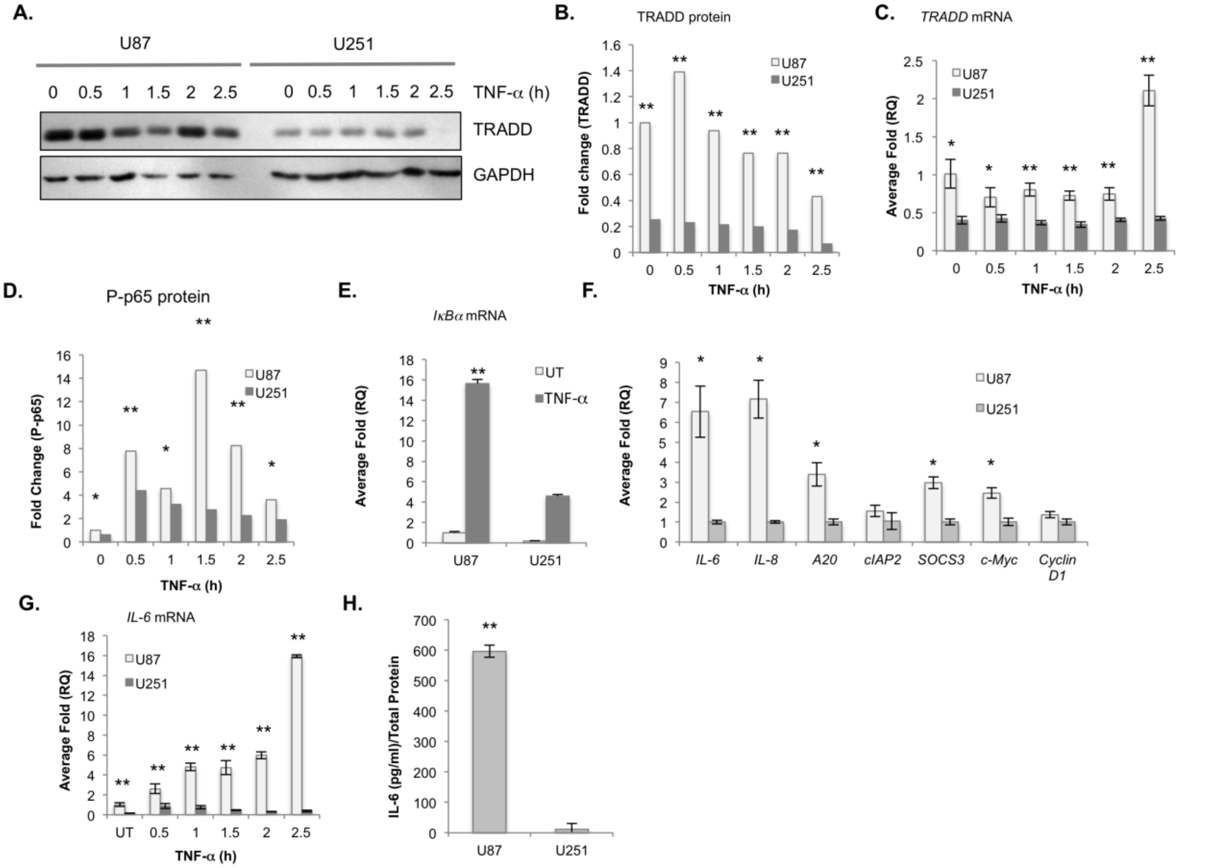

Supp. Fig. 3. MiR-31 Inversely Correlates with TRADD Levels and NF-κB Activities in Human Glioma Cells. A and B. The levels of TRADD protein were analyzed by immunoblotting **(A)** or by fluorescent densitometry **(B)**. (**, $p < 0.005$). **C, E, F and G.** The levels of *TRADD* **(C), *IκBα*** **(E)**, NF-κB regulated genes (*IL-6*, *IL-8, A20, cIAP2*, *SOCS3*, *c-Myc*, *Cyclin D1*) **(F)** and *IL-6* **(G)** mRNA levels were evaluated by qRT-PCR in the absence and presence of TNF-α stimulation. (*, $p < 0.05$; **, $p < 0.005$). **D.** Activated NF-κB p65 (P-p65) protein levels were analyzed by immunoblotting and fluorescent densitometry. (*, $p< 0.05$; **, $p< 0.005$). **H.** IL-6 protein levels were evaluated by ELISA. (**, $p < 0.005$).

p<0.005).

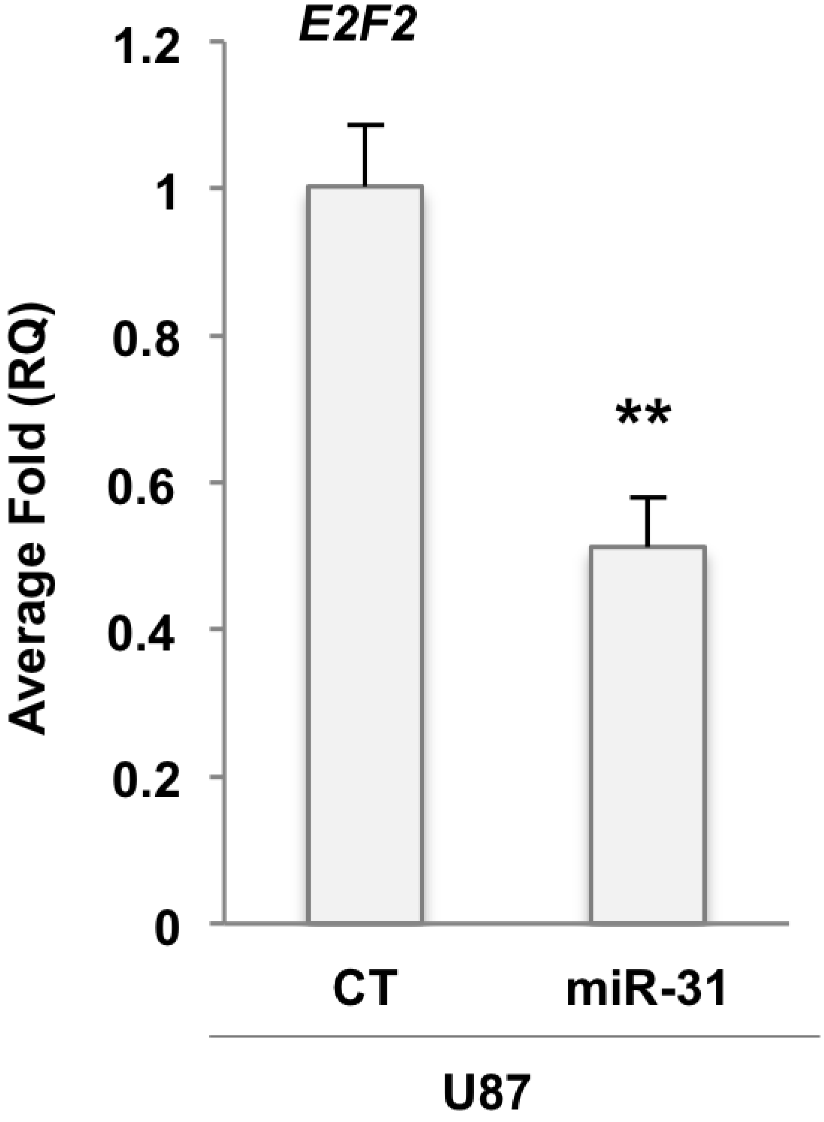

Supp. Fig. 4. MiR-31 Inhibits *E2F2* Expression, a Validated miR-31 Target Gene. Cells were grown as described in Fig. 3A, and *E2F2* mRNA levels analyzed by qRT-PCR. (**, p<0.005).

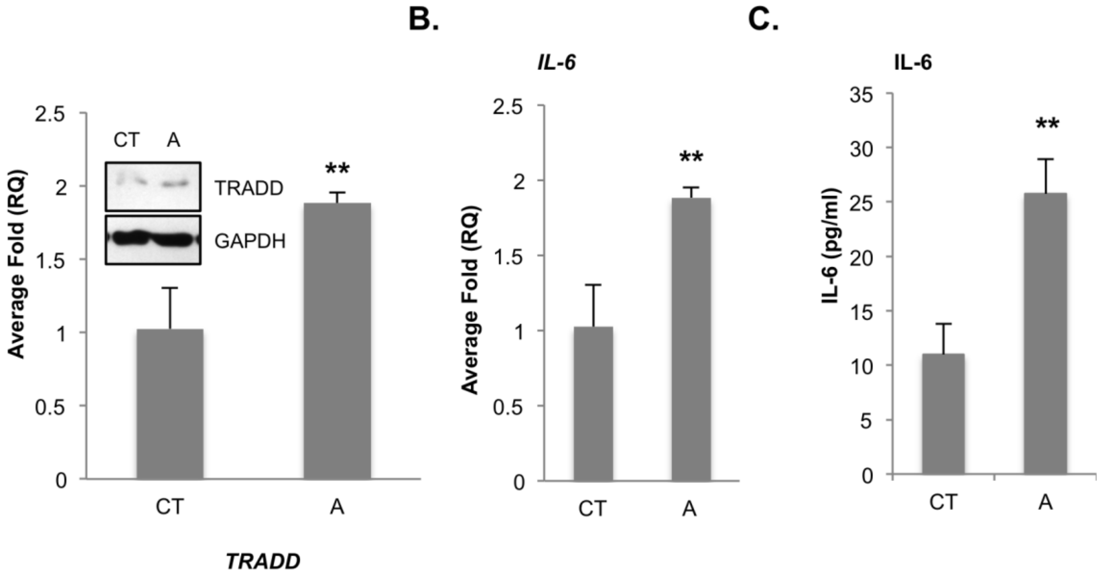

Supp. Fig. 5. Reductions in miR-31 Enhance TRADD Expression and NF-κB Activities. A-C. U251-MG cells were transfected with CT or AntagomiR-31 (A) for 48 h. **A.** The levels of TRADD mRNA and protein were measured by qRT-PCR and immunoblot analysis, respectively. (**, $p < 0.005$). **B, C.** The levels of IL-6 mRNA **(B)** and protein **(C)** were measured by qRT-PCR and ELISA, respectively. (**, $p < 0.005$).

A.

	MIR31HG$^{-/-}$	*MIR31HG*$^{+/+}$	
NFKBIA$^{+/-}$	39	128	167
NFKBIA$^{+/+}$	129	267	396
	168	395	563

Pearson uncorrected P value = 0.0029

B.

	CDKN2A$^{-/-}$	*CDKN2A*$^{+/+}$	
NFKBIA$^{+/-}$	103	64	167
NFKBIA$^{+/+}$	220	176	396
	323	240	563

Pearson uncorrected P value = 0.18

Supp. Fig. 6. *MIR31HG* and *NFKBIA* Deletions are Mutually Exclusive Events. Two-way contingency table analysis based on Pearson's chi-square test was used to assess patterns of potential mutual exclusivity of *NFKBIA* deletions and *MIR31HG* deletions **(A)** or *CDKN2A* deletions **(B)**. Odds ratios in the two-way contingency table analysis were computed according to the equation (a/b)/(c/d), where (for example) a and b indicate the number of *MIR31HG* deleted genes with and without *CDKN2A* deletion, respectively; and c and d indicate the number of *MIR31HG* wild-type genes with and without *CDKN2A* deletion, respectively, using Woolf's method for variance estimation. Confidence intervals for the estimated parameters were computed based on "constant chi-square boundaries" [47].

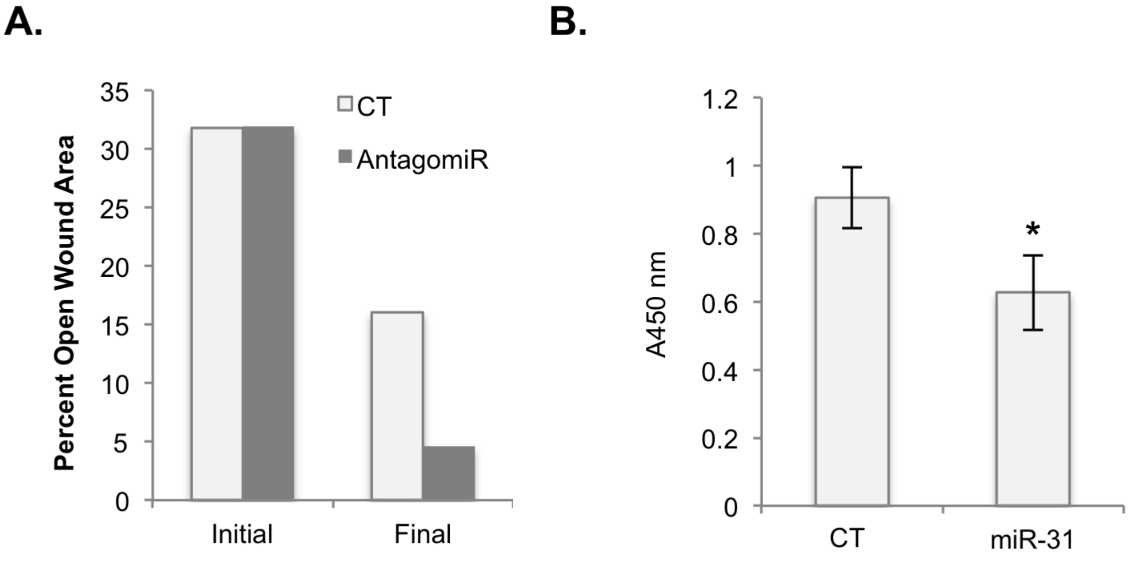

Supp. Fig. 7. MiR-31 Status Inversely Correlates with Glioma Cell Migration and Metabolic Activity. A. U251-MG miR-31 positive cells were transfected with control (CT) miRNA or miR-31 antagomiR (AntagomiR) and grown to confluence. Cells were wounded and allowed to recover for 8 h. Percent of open wound area was quantified using TScratch software. Data are shown as replicates of three; representative of two experiments. **B.** U87 cells were transfected with control miR (CT) or miR-31 and assessed using the WST-1 assay. Data are shown as replicates of four; representative of two experiments. (*, $p < 0.05$).

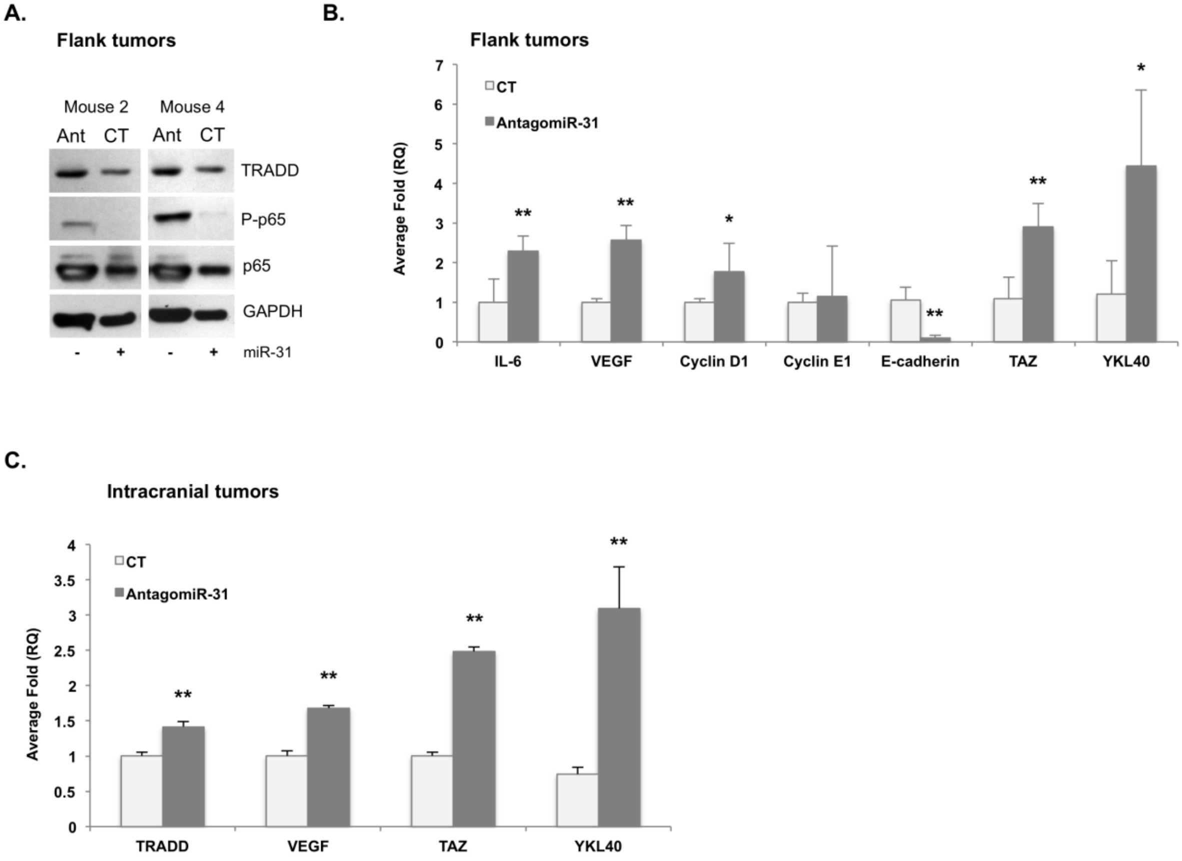

Supp. Fig. 8. The Levels of miR-31 Inversely Correlate with Tumor Growth *In Vivo*. A-D. JX12 PDGXs were transfected with CT or AntagomiR-31 and grown s.c. in the flank **(A, B)** or as intracranial tumors **(C, D)**. **A and B.** The levels of TRADD protein, P-p65 and p65 **(A)**, and *IL-6*, *VEGF*, *Cyclin D1*, *Cyclin E1*, *E-cadherin*, *TAZ* and *YKL40* **(B)** mRNA in resected flank tumors were analyzed by immunoblotting or qRT-PCR. (*, $p<0.05$; **, $p<0.005$). Results shown in **(B)** are pooled from 2 tumors each. **C.** At resection, intracranial tumors were evaluated by H&E **(C)** or qRT-PCR **(D)**. Results shown in **(D)** are pooled from 4 tumors each. (**, $p<0.005$).

A.

	U87	U251	Total
(-) miR-31	3/5	4/5	7/10
(+) miR-31	0/5	2/5	2/10

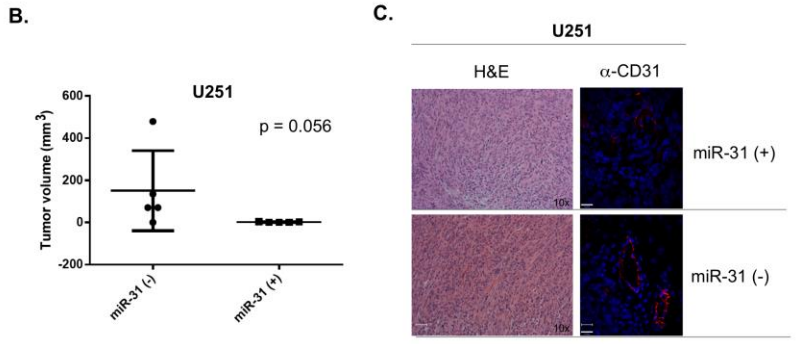

Supp. Fig. 9. The Levels of miR-31 Inversely Correlate with Tumor Growth *In Vivo*. A. U87 and U87/miR-31, or U251 and U251/AntagomiR-31 cells were grown in the flanks of nude mice and evaluated for tumor growth over an 80-day period. Incidents of tumor formation are presented. **B.** Tumor volume of U251 tumors. **C.** U251 tumors described in **(A, B)** were analyzed by IHC using H&E and antibodies specific for CD31 (x, y-scale of 21 μm).

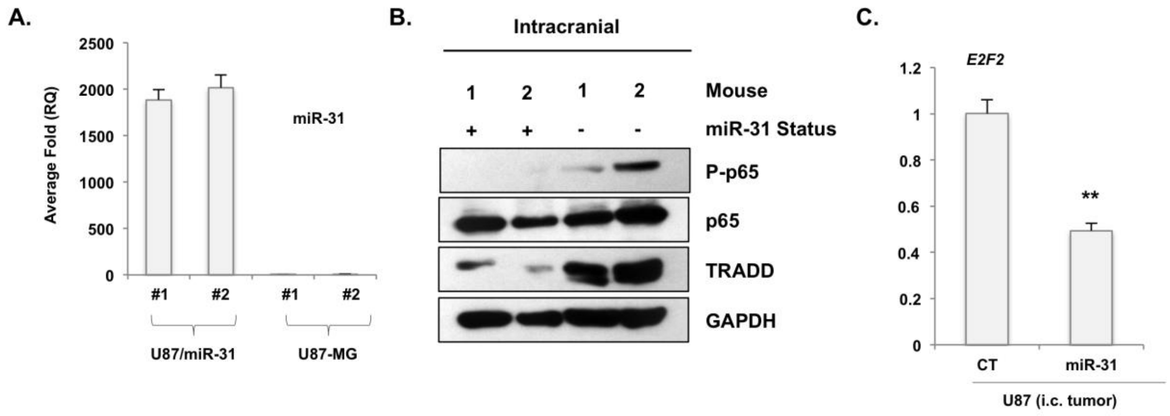

Supp. Fig. 10. The Levels of miR-31 Inversely Correlate with GBM Growth *In Vivo*. A-C. Resected tumors described in Fig. 6C were analyzed by qRT-PCR **(A, C)** or immunoblotting **(B)**.
